# Modifications to the NEATS instrument for more appropriate and reproducible assessment of guidelines trustworthiness

**DOI:** 10.1002/gin2.12025

**Published:** 2024-07-25

**Authors:** Maryam Ghadimi, Gordon Guyatt, Romina Brignardello‐Petersen

**Affiliations:** ^1^ Department of Health Research Methods, Evidence, and Impact McMaster University Hamilton Ontario Canada; ^2^ Department of Medicine McMaster University Hamilton Ontario Canada; ^3^ MAGIC Evidence Ecosystem Foundation Oslo Norway

**Keywords:** assessment of trustworthiness, clinical practice guidelines, IOM standards, methodological quality, modifications, NEATS instrument, reproducibility

## Abstract

**Rational:**

The National Guideline Clearinghouse Extent of Adherence to Trustworthy Standards (NEATS) instrument measures the adherence of clinical practice guidelines to the trustworthiness standards proposed by the Institute of Medicine. However, rather than trustworthiness or methodological quality, the NEATS instrument evaluates the quality of reporting in addressing the management of conflict of interest of guideline development group members, systematic review process and assessment of the certainty of the evidence, rating the strength of recommendations, and updating plans. Furthermore, the NEATS instrument instructions on rating items are sufficiently limited that they may compromise the reproducibility of the instrument.

**Methods (Modifications to the NEATS Instrument):**

In the context of a specific methodological research project, we developed modifications to the NEATS instrument that include modifying the wording of items to capture trustworthiness rather than reporting quality and creating an algorithm for all items that maps responses to a series of prompting questions and guides the user in arriving at a rating from 1 to 5 or yes, no, or unknown, as applicable.

**Implications:**

Modifications to the NEATS instrument present a structured and practical framework to assess the degree to which clinical practice guidelines are trustworthy, and they ensure that items evaluate trustworthiness and improve the reproducibility of assessments across a range of raters' level of expertise in guideline methodology.

## Key points

- National Guideline Clearinghouse Extent of Adherence to Trustworthy Standards (NEATS) instrument measures the adherence of guidelines to the trustworthiness standards proposed by the Institute of Medicine.
- NEATS instrument, for some domains, evaluates the quality of reporting rather than trustworthiness or methodological quality and lacks sufficient instructions on rating items.
- We developed modifications to the NEATS instrument to address these limitations. We modified the wording of items to capture trustworthiness rather than reporting quality and created an algorithm for all items that guides the user on arriving at a specific rating.
- Our modifications ensure items evaluate the appropriateness of the guideline development process and improve the reproducibility of assessments across a range of raters' level of expertise in guideline methodology.

## 1 RATIONALE

Clinical practice guidelines are documents developed by a panel of individuals who, through systematic reviews of evidence, evaluate the benefits and harms of alternative care options to make recommendations,^1^ that are fundamental to clinical, public health, and policy decision‐making. It is, therefore, crucial to determine the extent to which users can have confidence in recommendations from guidelines. Multiple instruments are available to assess the trustworthiness of guidelines. The Appraisal of Guidelines for Research and Evaluation (AGREE) II instrument is the widely accepted one that has a comprehensive scope and intends to evaluate the rigour of methods, quality of reporting, and applicability of guidelines.^2^ The AGREE II, however, does not address important details about the process for conducting systematic reviews of evidence to inform the guidelines. The National Guideline Clearinghouse (NGC) Extent of Adherence to Trustworthy Standards (NEATS) instrument^3^ overcomes this limitation by translating the eight trustworthiness standards proposed by the Institute of Medicine (IOM)^1^ into 15 items with the aim to measure the methodological quality of guidelines—from which three are specific to the systematic review process. Box 1 presents the IOM standards for developing trustworthy clinical practice guidelines.

Box 1 **Institute of Medicine standards for developing trustworthy clinical practice guidelines.**

1. The processes by which a guideline is funded should be detailed explicitly and publicly accessible.
2. The guideline development group members should declare all current and planned financial and nonfinancial activities pertinent to the potential scope of the guideline.
  a) The chair or cochairs should not have a conflict of interest.
  b) Members with a conflict of interest should represent not more than a minority of the guideline development group.
  c) Members of the guideline development group should divest themselves of financial activities they or their family members have in entities whose interests could be affected by guideline recommendations.
  d) Funders should have no role in guideline development.
3. The guideline development group should be multidisciplinary and balanced, comprising a variety of methodological experts, clinicians, and populations expected to be affected by the guideline.
4. Guideline developers should use systematic reviews that meet standards set by the Institute of Medicine's Committee on Standards for Systematic Reviews of Comparative Effectiveness Research.
5. For each recommendation, guidelines should provide a rating of the strength of the recommendation based on consideration of (a) balance of benefits and harms; (b) certainty of the evidence underpinning the recommendation; and (c) value and preferences of patients.
6. Recommendations should be articulated in a standardized form detailing precisely what the recommended action is, and under what circumstances it should be performed.
7. External reviewers should comprise a full spectrum of relevant stakeholders, including scientific and clinical experts, relevant organizations, and patient representatives and a draft of the guideline at the external review stage or immediately following it should be made available to the public for comment.
8. The proposed date for future update should be documented in the guideline.
  a) Literature should be monitored regularly following the publication to identify the emergence of new, potentially relevant evidence and to evaluate the continued validity of the guideline.
  b) Guidelines should be updated when new evidence suggests the need for modification of recommendations.

When conducting a research project with the aim of addressing the trustworthiness of guidelines and to what extent trustworthiness has changed since the introduction of the IOM standards, we used the NEATS instrument to assess trustworthiness. In doing so, we faced two challenges. First, for some domains, the NEATS instrument evaluates the quality of reporting rather than the risk of bias or methodological quality, the latter being both the apparent intent of the instrument and a construct more relevant to guideline trustworthiness. For example, in addressing the search strategy from the systematic review supporting the guideline, the NEATS instrument asks whether the guideline or a companion document reports a list of databases, search terms, and time of the search. This question addresses reporting and not trustworthiness: authors may, for instance, report that they used only a single database, inadequate search terms, and the search may be outdated. They would satisfy the item in terms of reporting but not in terms of trustworthiness. Second, the instructions provided for using the instrument have limitations. For several items rated on a 5‐point Likert scale, for instance, users must determine how to assign ratings of ‘poor’, ‘fair’, ‘good’, ‘very good’, or ‘excellent’ without details about what makes adherence to an item fit into each of these categories. This threatens the reproducibility of the assessments and may lead to inconsistent judgements.

## 2 MODIFICATIONS TO THE NEATS INSTRUMENT

To optimize our ongoing research project, over multiple discussions, we reached a consensus on how to modify the instrument with two goals: (1) specifically addressing the appropriateness of the guideline development process and (2) improving the reproducibility of assessments across a range of raters' level of expertise in guideline methodology.

To achieve these goals, we first modified the wording of some items to capture trustworthiness rather than reporting quality. This includes items addressing whether the guideline panel appropriately managed the conflict of interests (COI) of members (item 2)^4^, ^5^, ^6^; whether the systematic search of available evidence was relevant, up to date, and included an adequate selection of databases and additional methods to identify relevant studies (item 5a)^7^, ^8^, ^9^; whether eligibility criteria of systematic review match recommendation questions and the study selection process was at low risk of bias (item 5b)^7^, ^8^, ^9^; whether assessment of the certainty of the evidence underlying recommendations was appropriate (item 6)^7^; whether the strength of recommendations matches balance of benefits and harms, values and preferences of patients, and certainty of underlying evidence (item 9)^10^; and whether the updating plan of the guideline is appropriate (item 12) (Table 1).

**Table 1:** Comparison of the wording of the National Guideline Clearinghouse Extent of Adherence to Trustworthy Standards (NEATS) items and modified items.

NEATS items	Modified items
**Item 2:** Financial conflicts of interest of guideline development group members have been disclosed and managed.	**Item 2:** Financial conflicts of interest of guideline development group members have been disclosed and appropriately managed.
**Item 5a:** The guideline or a related companion document describes a search strategy that includes a listing of database(s) searched, a summary of search terms used, and the specific time period covered by the literature search.	**Item 4a:** The guideline or related companion documents describe a search strategy that matches the relevant recommendation questions, includes an adequate selection of databases and additional methods to identify relevant studies, and is up to date.
**Item 5b:** The guideline or a related companion document describes the study selection that includes the number of studies identified, the number of studies included, and a summary of inclusion and exclusion criteria.	**Item 4b:** The guideline or related companion documents describe the study selection process that includes unambiguous eligibility criteria that match the relevant recommendation questions, number of studies retrieved from the search, duplicate screening of identified studies, number of studies retrieved from the search number of studies included, and reasons for excluding studies.
**Item 6:** The guideline provides a grading or rating of the level of confidence in or certainty regarding the quality or strength of the evidence for each recommendation.	**Item 4c:** The guideline or related companion documents provide a synthesis of evidence from the included studies and an appropriately assessed the certainty of evidence for each recommendation.
**Item 9:** The guideline gives a rating of the strength of the recommendation for each recommendation that takes into account benefits and harms, available evidence, and confidence in the underlying evidence.	**Item 5:** The guideline gives a rating of the strength of the recommendation for each recommendation that was appropriately informed by the balance of benefits and harms of alternative care options, value and preferences of patients, and certainty of the underlying evidence.
**Item 12:** The guideline describes a procedure to update the guideline.	**Item 8:** The guideline or supporting documents provide an adequate time frame for review of evidence for potential updating and describe adequate criteria that warrant updating the recommendation.

**Figure 1:**
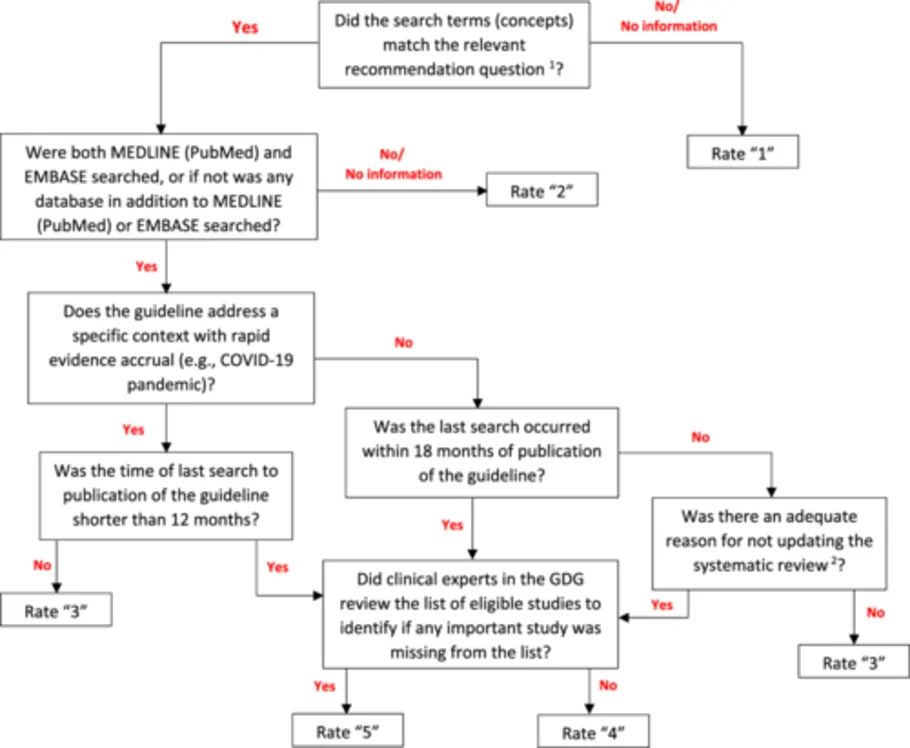
The algorithm for the assessment of the search strategy of the systematic review of evidence informing the guidelines (item 4a).

Second, we created an algorithm for all items that maps the responses to a series of prompting questions and guides the user on arriving at a rating from 1 to 5 or yes, no, or unknown, as applicable. Figure 1 presents an example of the algorithm. Where applicable, we provide additional details, including examples, to interpret the prompting questions. The prompting questions were answered ‘Yes’, ‘No’ or ‘No information/unclear’ and were agreed on for less subjectivity in the interpretation. All algorithms start with a key question where the answer ‘No’ or ‘No information/unclear’ means the lowest adherence to the given item.

Additionally, we grouped items from the NEATS instrument addressing the same aspect of the guideline development into a single item. For example, we grouped together three items (items 3a, 3b and 4, addressing the inclusion of clinical experts, methodologists, and patients' representatives) related to the ‘composition of guideline development group’. Another example is the item on ‘rating the certainty of the evidence’ (item 6) which we considered as a relevant aspect of the ‘synthesis of evidence’ item (item 5c). We also grouped together items 7–9 to address the appropriateness of the rating of strength of recommendations.

The Supporting Information S1 provides the modified instrument manual. Our modified tool includes 10 items covering all eight domains addressed in the NEATS instrument. Two of the 10 items are measured using two or three response options (one item [yes/no] and one item [yes/no/unknown]), and 8 items are rated on the 5‐point Likert scale. The response ‘Yes’ on categorical items or a score of 5 on the other items represents the highest adherence to the given item, and a score of 4 represents acceptable adherence. We agreed on the following items as critical in addressing the trustworthiness of clinical practice guidelines: disclosure and management of COIs (item 2), study selection and synthesis of evidence from the systematic review process (items 4b and 4c), and grading the strength of recommendations (item 5).

## 3 IMPLICATIONS

Our modification of the NEATS instrument presents a structured framework to evaluate the degree to which practice guidelines are trustworthy. Because we developed this modification to address a specific need that arose in the context of a research project, this modification has not undergone formal instrument development or validation. In addition, to ensure feasibility, we decided to simplify the assessment of some of the items (e.g., we focused on financial COIs, usually classified as high levels of COI, and did not include intellectual COIs that are considered moderate^11^). Nevertheless, this modification raises awareness regarding the limitations of the NEATS instrument and provides a promising practical solution to addressing those limitations. Researchers who want to study the trustworthiness of guidelines, journal editors who want to evaluate the trustworthiness of guidelines for publication, and clinicians who want to recognize a trustworthy guideline for use in their practice may benefit from our modification to the NEATS instrument that facilitates much more explicit and appropriate judgements.

## AUTHOR CONTRIBUTIONS

**Maryam Ghadimi**: Conceptualization; methodology; investigation; project administration; validation; visualization; writing—original draft preparation; writing—review and editing. **Gordon Guyatt**: Conceptualization; methodology; validation; writing—review and editing. **Romina Brignardello‐Petersen**: Conceptualization; methodology; supervision; validation; writing—review and editing.

## CONFLICT OF INTEREST STATEMENT

The authors declare no conflict of interest.

## ETHICS STATEMENT

Not applicable.

## Supporting information

Supporting information.

## Data Availability

Data sharing is not applicable to this article as no new data were created or analysed in this study.
