# Supplementary material for: Modifications to the NEATS instrument for more appropriate and reproducible assessment of guidelines trustworthiness
Source: Clin Public Health Guidel. 2024 Jul 25;1(3):e12025. doi: 10.1002/gin2.12025 (PMC13563409; doi:10.1002/gin2.12025)
Supplement: Supplementary file 1 [file GIN2-1-e12025-s001.pdf]

A guideline trustworthiness assessment tool based on the NEATS Instrument – Instruction Manual

Please read carefully:

- This tool includes 10 items, of which 2 items are measured using categorical response options (one item (yes / no) and one item (yes/ no/ unknown)) and 8 items are rated on the 5-point Likert scale.
- Each item should be judged using the provided algorithm. Please read the footnotes (if provided) for more information about the prompting questions in the algorithm.
- For each item, you will find information on "where to look" to find relevant data for your judgment.
- The assessment of trustworthiness can be performed at the guideline level (i.e., assessing all recommendations in a guideline) or at the recommendation level (i.e., assessing a single or a group of recommendation(s) of interest in a guideline). Therefore, the user should prespecify the assessment level and apply the tool accordingly.

The definitions you should be familiar with before starting your assessment:

- Clinical practice guideline (CPG): Documents that were developed by a formal panel of individuals who evaluate systematic reviews of evidence and benefits and harms of alternative care options to make recommendations.
- Recommendation: An actionable statement that describes a course of action recommended or suggested by the guideline based on clinical questions, evidence retrieval, and consideration of other information in an analytic framework.
- Guideline development group (GDG) (may also be labelled as writing committee, task force, or guideline panel): A group of individuals who decides on topics to be covered within the guideline, formulates questions, develops, and agrees on the recommendations in the guideline using evidence summaries prepared by a working group, and endorses the final guideline document for approval by the oversight committee.
- Certainty of the evidence (may also be labelled as quality of evidence or level of evidence): The level of confidence in the estimates of the effect of an intervention versus a comparator for a specific outcome. There are a variety of systems for evaluating the certainty of evidence which consider different characteristics of the evidence and use different categories of certainty.
- Strength of recommendations (may also be labelled as class/level of recommendation): The degree to which the GDG is confident in the balance between the desirable and undesirable consequences of implementing the recommendation. There are different frameworks for grading the strength of recommendation. These frameworks consider different criteria in determining the strength of recommendation and label the level of strength differently.
- Values and preferences of patients: The relative importance patients place on the health outcomes. This implicitly relates to other aspects that are relevant to a decision (e.g., equity, feasibility, or acceptability considerations).

1. Disclosure of Guideline Funding Source

The clinical practice guideline discloses and states explicitly its funding source(s).

Where to Look: Examine the methods for guideline development or guideline development process section, acknowledgement section, appendices, or link to any publication platform (like MAGICapp, GRADEpro, or any website, etc.) for "funding source(s)".

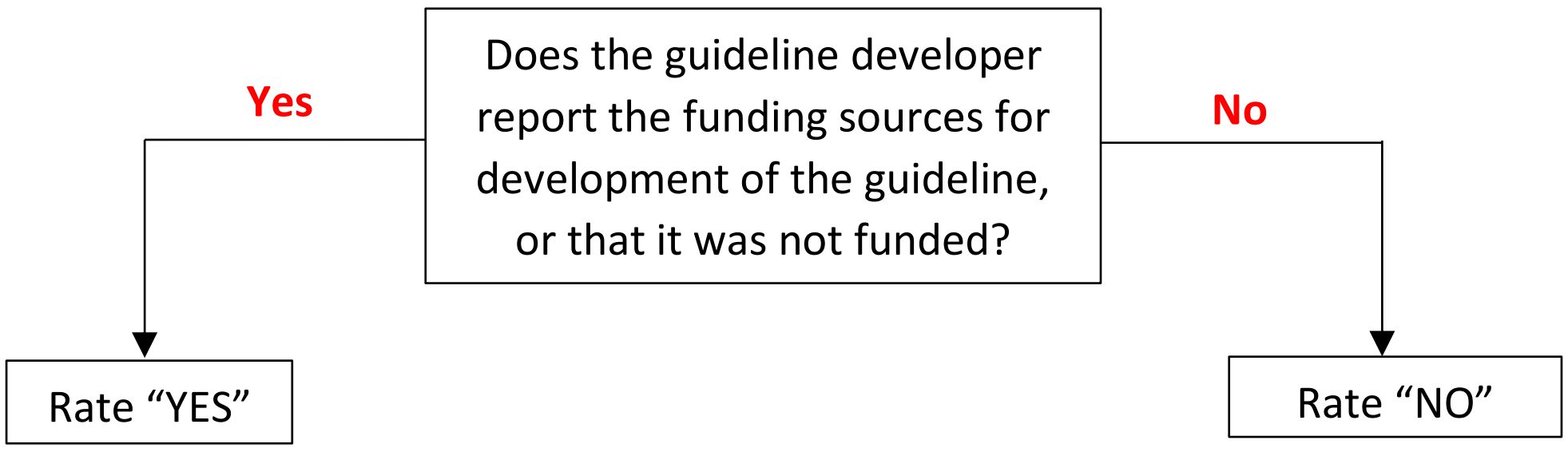

2. Disclosure and management of conflict of interests (COIs)

Financial COIs of guideline development group members have been disclosed and appropriately managed [1].

Where to Look: Examine the methods for guideline development or guideline development process section, authorship section, appendices, or link to any publication platform (like MAGICapp, GRADEpro, or any website, etc.) for “conflict of interests” or “competing interests” or “disclosure” or “declaration of interests”.

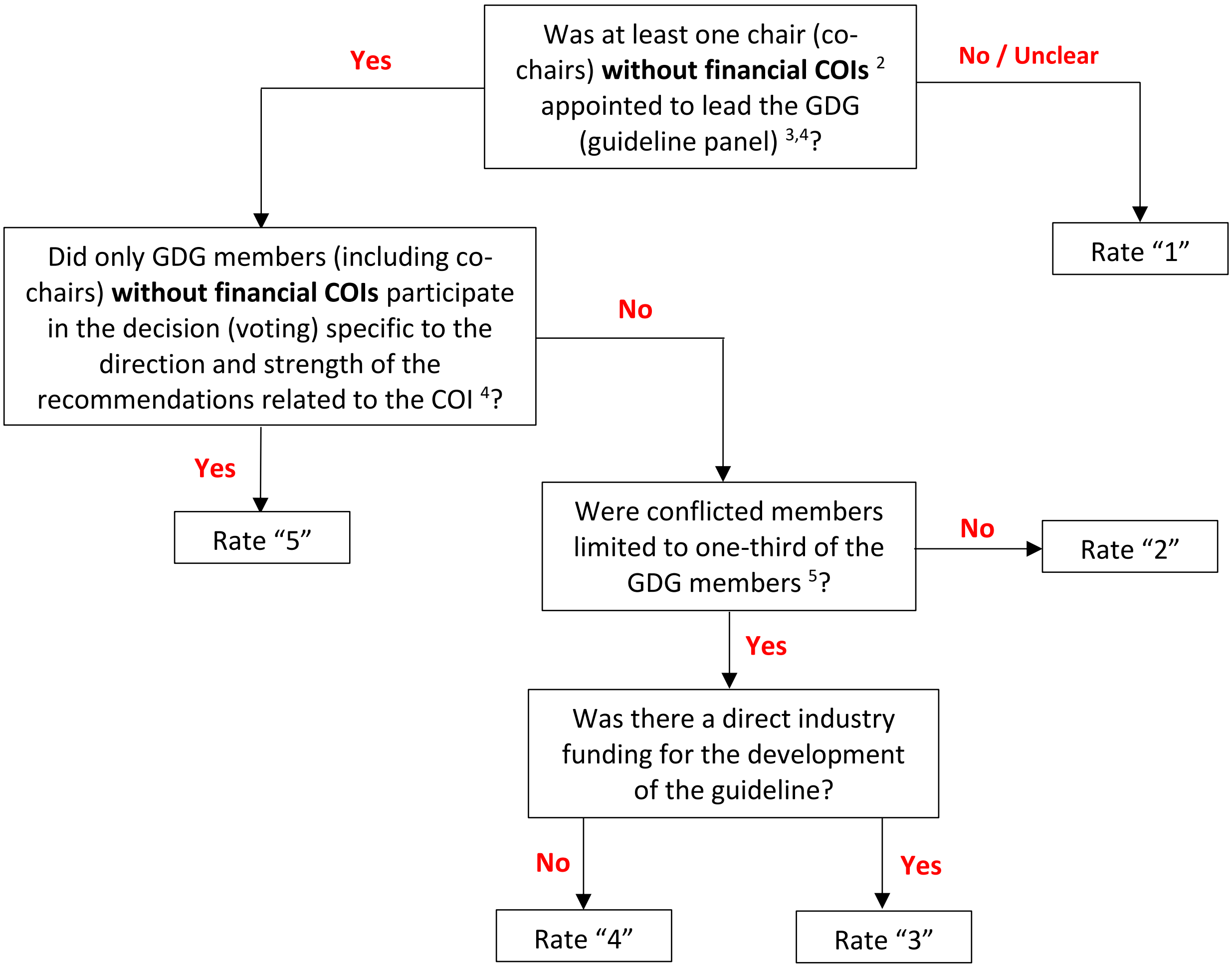

[1] This algorithm only addresses high level of COIs (financial COIs) according to the classification proposed by the American College of Physicians Clinical Guidelines Committee (DOI: https://doi.org/10.7326/M18-3279). Users can also assess intellectual COIs refers to “academic activities that create the potential for an attachment to a specific point of view that could unduly affect an individual’s judgment about a specific recommendation”. Users can use the same algorithm to assess guidelines concerning intellectual COIs.

[2] Financial COIs includes any financial benefit (e.g., any income from employment, consulting, speaking engagements, advising, and other management activities; equity ownership in public and privately traded companies; honoraria; loans; royalties, patents, contracts, and grants; and expert testimony) received from **for-profit-organizations** (e.g., pharmaceutical companies) <u>related to the topic discussed or products</u>

recommended in the guideline. Please note, funding received from governmental organizations (e.g., CIHR, NIH) should not be considered financial COI.

[3] If no information about COI is provided in the guidelines, choose "Unclear" and rate "1".

[4] Guidelines, in the conflict-of-interest section, may explicitly state that "no member has financial COIs" or "declared financial interests of the member perceived not in conflict with guideline topics or perceived nonsignificant that requires no action" or "members with financial COIs were excluded from participation in the voting of relevant recommendations", in any of these cases, choose "YES". If none applied, please review the COI declarations of each member.

[5] Guidelines may list GDG members for each recommendation rather than at the guideline level.

3. Guideline Development Group (GDG) Composition: Multidisciplinary

The GDG includes relevant clinical experts, methodologist(s), and patient representative(s).

Where to Look: Examine the opening paragraphs, methods for guideline development or guideline development process section, authorship section, acknowledgement section, contribution section, appendices, or link to any publication platform (like MAGICapp, GRADEpro, or any website, etc.) for the "composition of GDG".

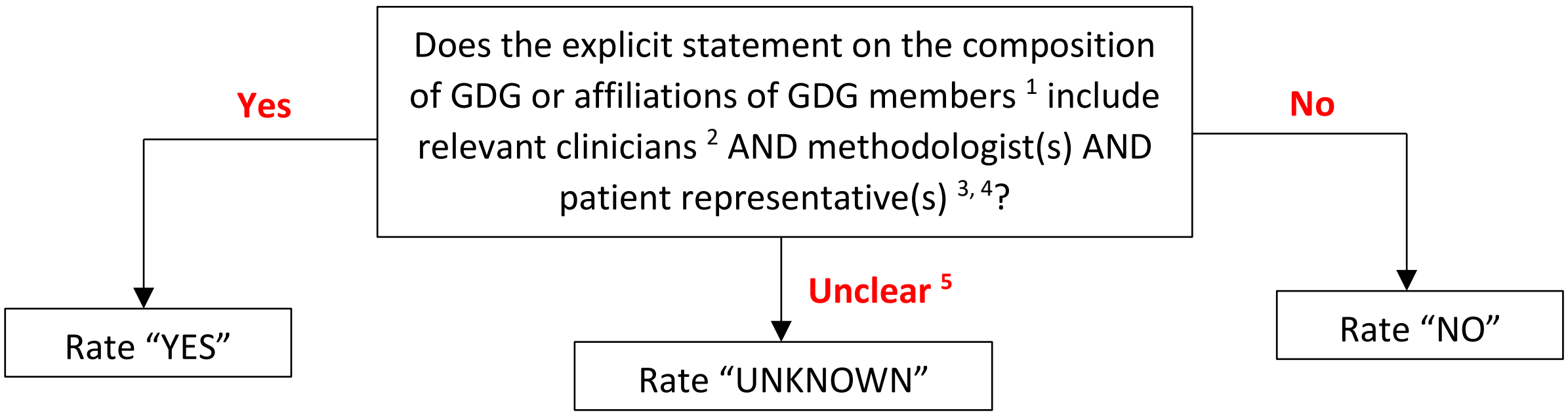

[1] Please note that the GDG may be identified as "writing committee", "task force", or "guideline panel". If the guideline did not identify the GDG, writing committee, task force, or guideline panel (which is rare), please consider the authors of the guideline as members of the GDG. Please note that you do not need to check the affiliations of all members; when you identified all 3 groups (clinicians, methodologist(s), patient representative(s)), you can stop reviewing the affiliations of remaining members and choose "YES" for this question.

[2] Relevant clinicians are individuals with clinical specialties relevant to the topic of the guideline.

[3] Patient representatives include current and former patients, carers who provide non-reimbursed care and support to patients including family members, members of the public as potential patients and as funders of healthcare through taxation, or advocates from patient/consumer organizations. The inclusion of one of these categories in the GDG is adequate to ensure that the perspective of patients was included in the guideline development.

[4] Guidelines may list GDG members for each recommendation rather than at the guideline level.

[5] Affiliations are not sufficiently specified that prevent from confirming whether the GDG included all 3 disciplines, for example, you reviewed affiliations of all members and confirmed that clinicians (e.g., affiliations: Department of Medicine, McMaster University, Hamilton, Ontario, Canada; Division of General Internal Medicine, University Hospitals of Geneva, Geneva, Switzerland; St. George's University Hospital, UK; Hospital Universitario Valle Hebron, Spain) and patient representative (e.g, World Hepatitis Alliance, United Kingdom) were included but the affiliations of other member are too broad (University of Sao Paulo, Brazil; University of North Carolina, USA) to be able to confirm if they were methodologists or belong to any other groups.

4a. Use of a Systematic Review of Evidence– Search strategy

The guideline or related companion documents describe a search strategy that matches the relevant recommendation questions, includes an adequate selection of databases and additional methods to identify relevant studies, and is up to date.

Where to Look: Examine methods for guideline development or guideline development process section, appendices or any publication platform (like MAGICapp, GRADEpro, or any website, etc.) for information on the "methods of literature search". In the methods for guideline development or guideline development process section, guidelines may refer to existing systematic review(s) that informed the guideline. In this case, you need to find the full text of the linked systematic review(s) and examine the search strategy presented in the systematic review publication. Sometimes guidelines may explicitly refer to a method guidance for the development of guidelines or conducting systematic reviews of evidence in the guideline development section and provide insufficient information on the search strategy in the guideline and supporting documents. In this case, you should find the full text of the guidance and check the search strategy to see if relevant information is provided.

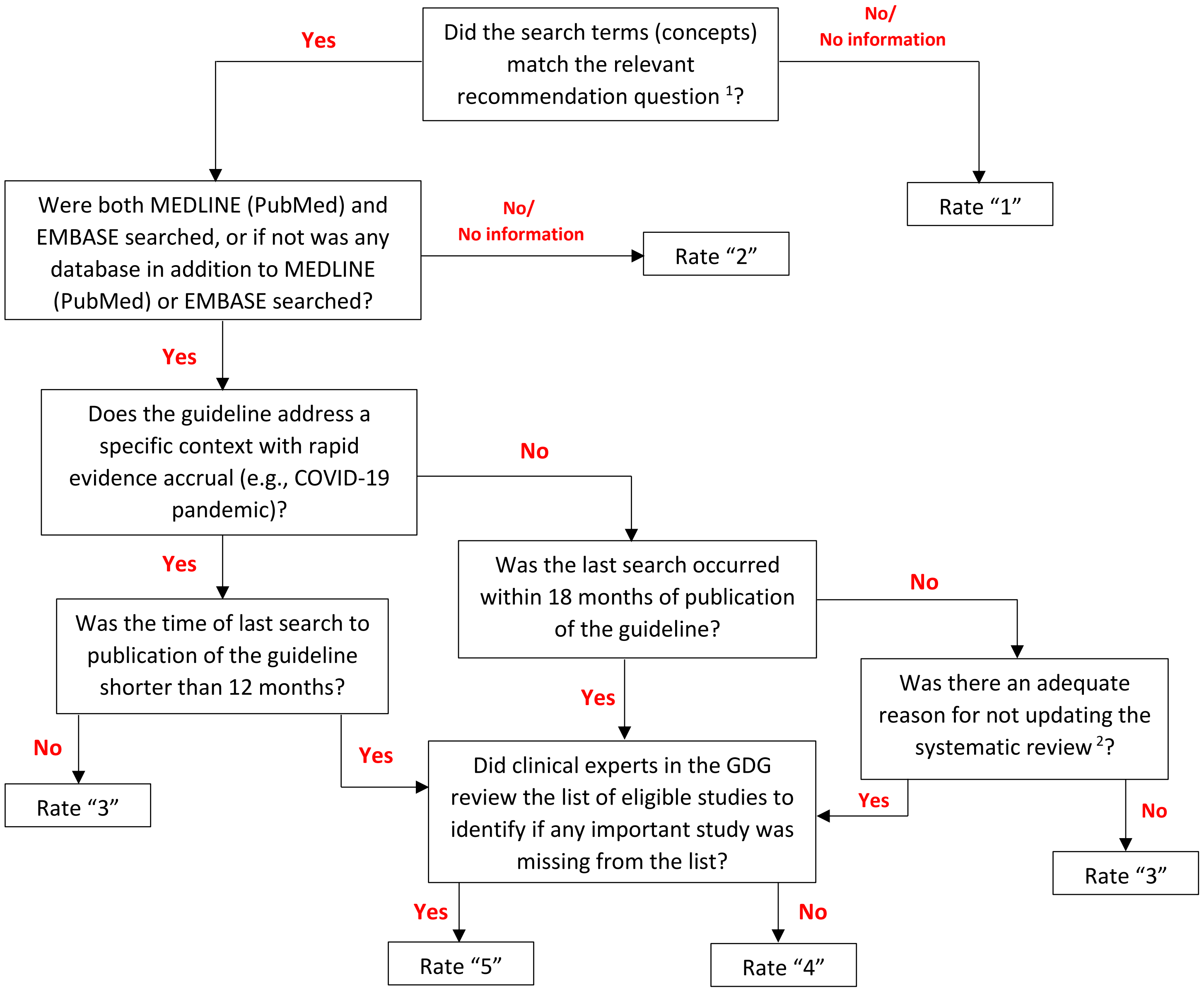

[1] This question does not ask if the search strategy included all PICO components, rather it aims to evaluate whether any search term (concept) that was included in the search strategy matches the related component in the research question.

[2] An adequate reason may relate to the activity in the clinical topic, availability of new evidence, influence of new evidence on the conclusion of the systematic review, etc.

4b. Use of a Systematic Review of Evidence – Study selection

The guideline or related companion documents describe the study selection process that includes unambiguous eligibility criteria that match the relevant recommendation questions, number of studies retrieved from the search, duplicate screening of identified studies, number of studies included, and reasons for exclusion of studies.

Where to Look: Examine methods for guideline development or guideline development process section, appendices or any publication platform (like MAGICapp, GRADEpro, or any website, etc.) for information on "eligibility criteria and study selection process". In the methods for guideline development or guideline development process section, guidelines may refer to existing systematic review(s) that informed the guideline. In this case, you need to find the full text of the linked systematic review(s) and examine the eligibility criteria and study selection process presented in the systematic review publication. Sometimes guidelines may explicitly refer to a method guidance for the development of guidelines or conducting systematic reviews of evidence in the guideline development section and provide insufffiecient information on the study selection in the guideline and supporting documents. In this case, you should find the full text of the guidance and check the study selection process to see if relevant information is provided.

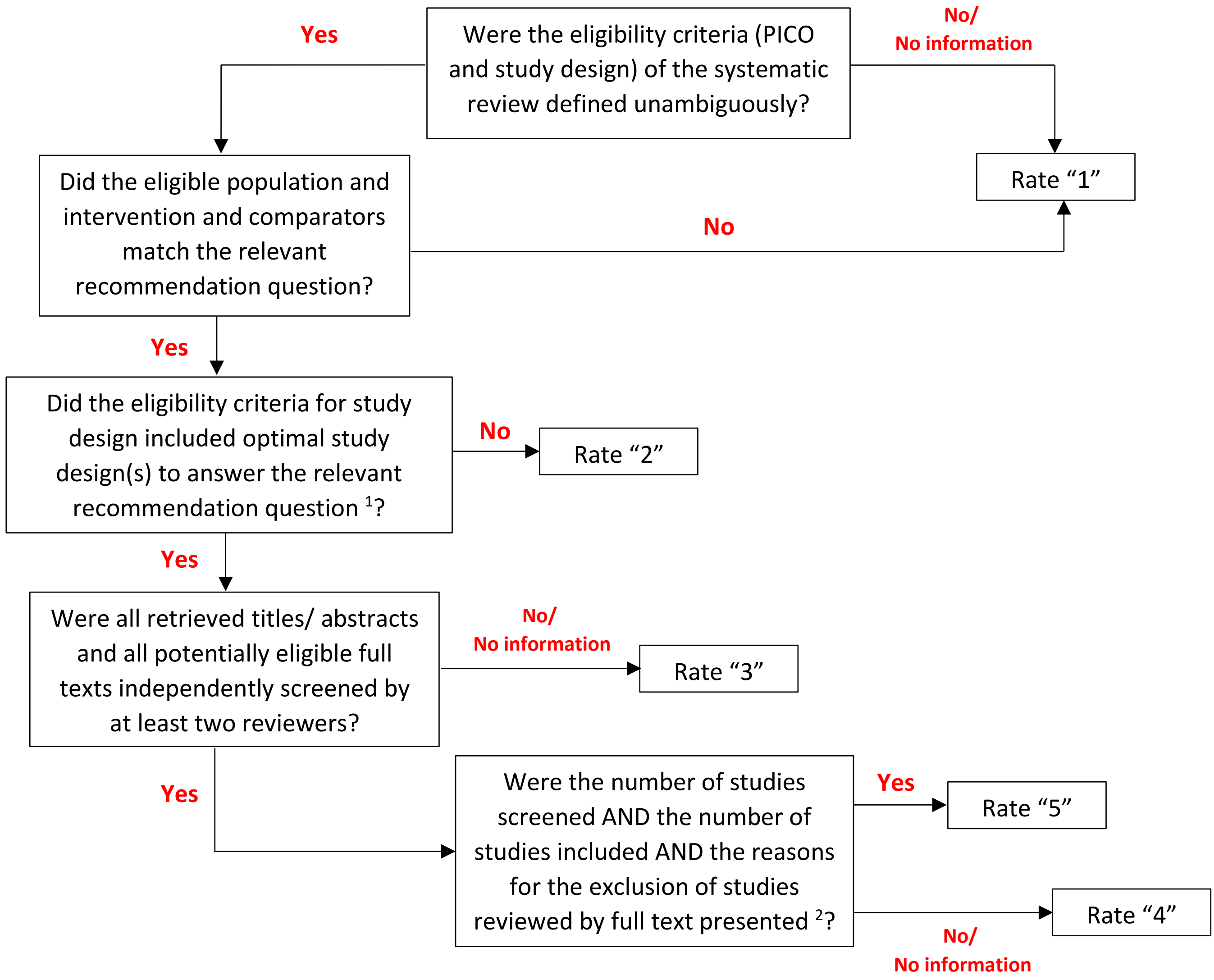

[1] Please note this question asks about the eligibility criteria, not the study designs that were finally included in the systematic review.

[2] Reasons for exclusion of studies could be presented as 1) a table in which each of the excluded studies and the reason for exclusion are listed; 2) cumulative reasons of exclusion with references to all of the studies excluded for each specific reason.

4c. Use of a Systematic Review of Evidence – Synthesis of evidence

The guideline or related companion documents provide a synthesis of evidence from the included studies and an appropriately assessed certainty of evidence for each recommendation.

Where to Look: First, please examine recommendations in the guideline main document, appendices, and any platform (like MAGICapp, GRADEpro, or any website, etc.) to address relevant questions from the algorithm (the first 3 boxes), then you should examine methods for guideline development or guideline development process section, any methods guidance that referred to, or the linked existing systematic review that you picked for item 4a/b (if relevant) to check the appropriateness of assessment of the certainty of evidence (the last 2 boxes).

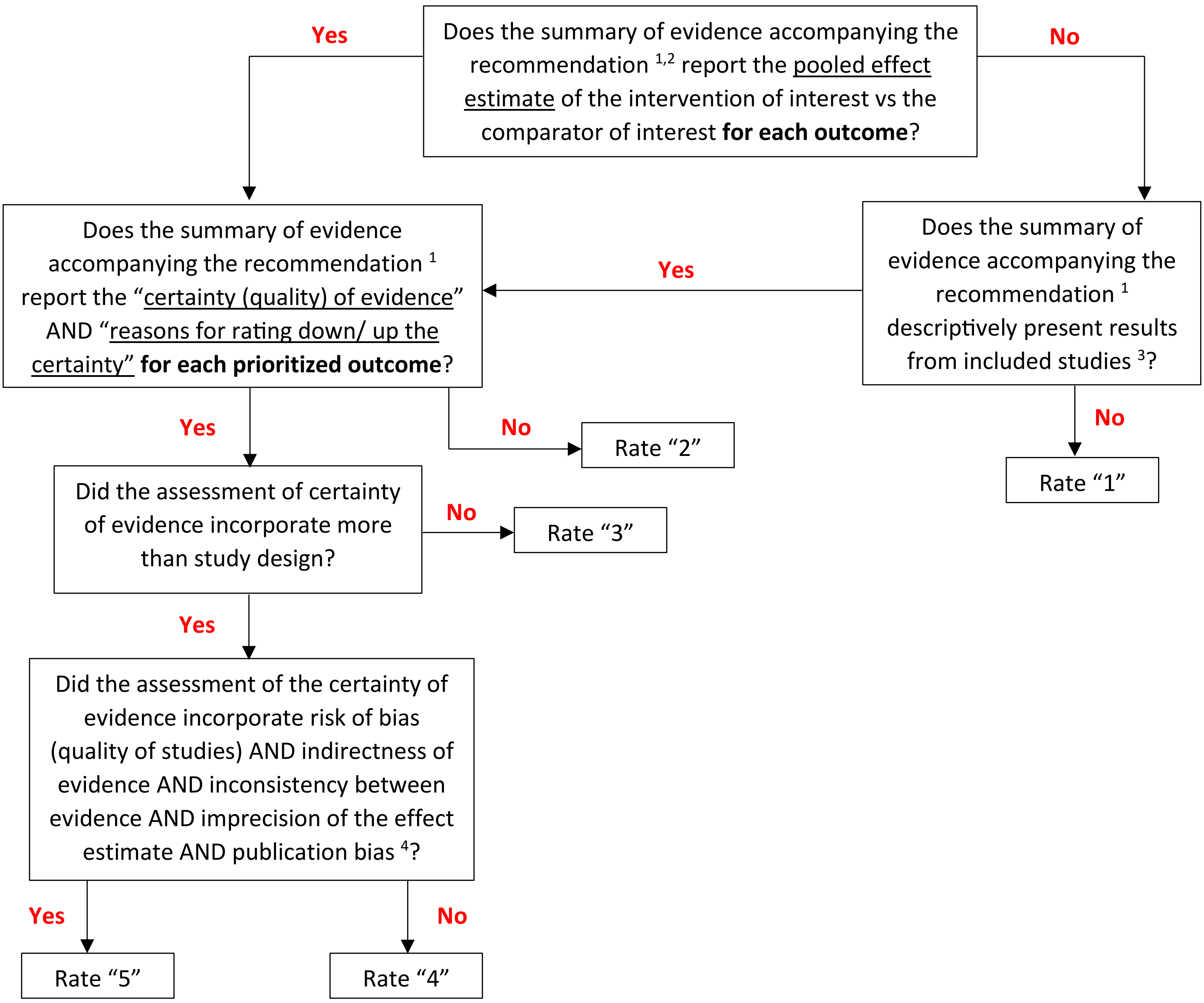

[1] Please note sometimes guidelines provide detailed evidence summary in the evidence summary table (in the supporting document or publication platforms) and evidence summary text linked to the recommendation in the main guideline documents lacks the required information. Therefore, you need to check all the sections mentioned in the "where to look" before finalizing your answer to this question.

[2] Please note each recommendation must be linked to a specific summary of evidence, so if you see that the guideline presents a group of recommendations followed by the summary of evidence without a clear linkage between each recommendation with a specific part of the summary of evidence, please rate "1".

[3] The descriptive presentation of findings from included studies can be reported either for each study (usually in tables in the supplementary file) or across studies per outcome (e.g., most studies showed a trivial reduction in mortality or no effect.).

[4] Please note domains of the certainty assessment might be labelled differently in different frameworks. The following explanation of each domain would help you to identify if it is incorporated in the assessment of the certainty of evidence. **Risk of bias** refers to the extent to which flaws in the design and execution of studies (e.g., sequence generation, allocation concealment, blinding, incomplete data, selective reporting of outcomes, confounding, etc.) could bias the estimate of effect for each outcome; **indirectness** refers to when population, intervention, or outcomes in included studies differs from the eligibility criteria of the systematic review; **inconsistency (heterogeneity)** refers to the degree of variability in effect size of an intervention on a specific outcome between included studies; **imprecision** refers to the degree of variability around effect estimate for a specific outcome; **publication bias (reporting bias)** refers to inclusion of unrepresentative sample of studies due to the absence of results from studies that should have been included in the synthesis (whether the studies not included are published or not).

5. Grading the strength of recommendation

The guideline gives a rating of the strength of the recommendation for each recommendation that was appropriately informed by the balance of benefits and harms of alternative care options, value and preferences of patients, and certainty of the underlying evidence.

Where to Look: Examine recommendations and accompanying summary of evidence as described in each box.

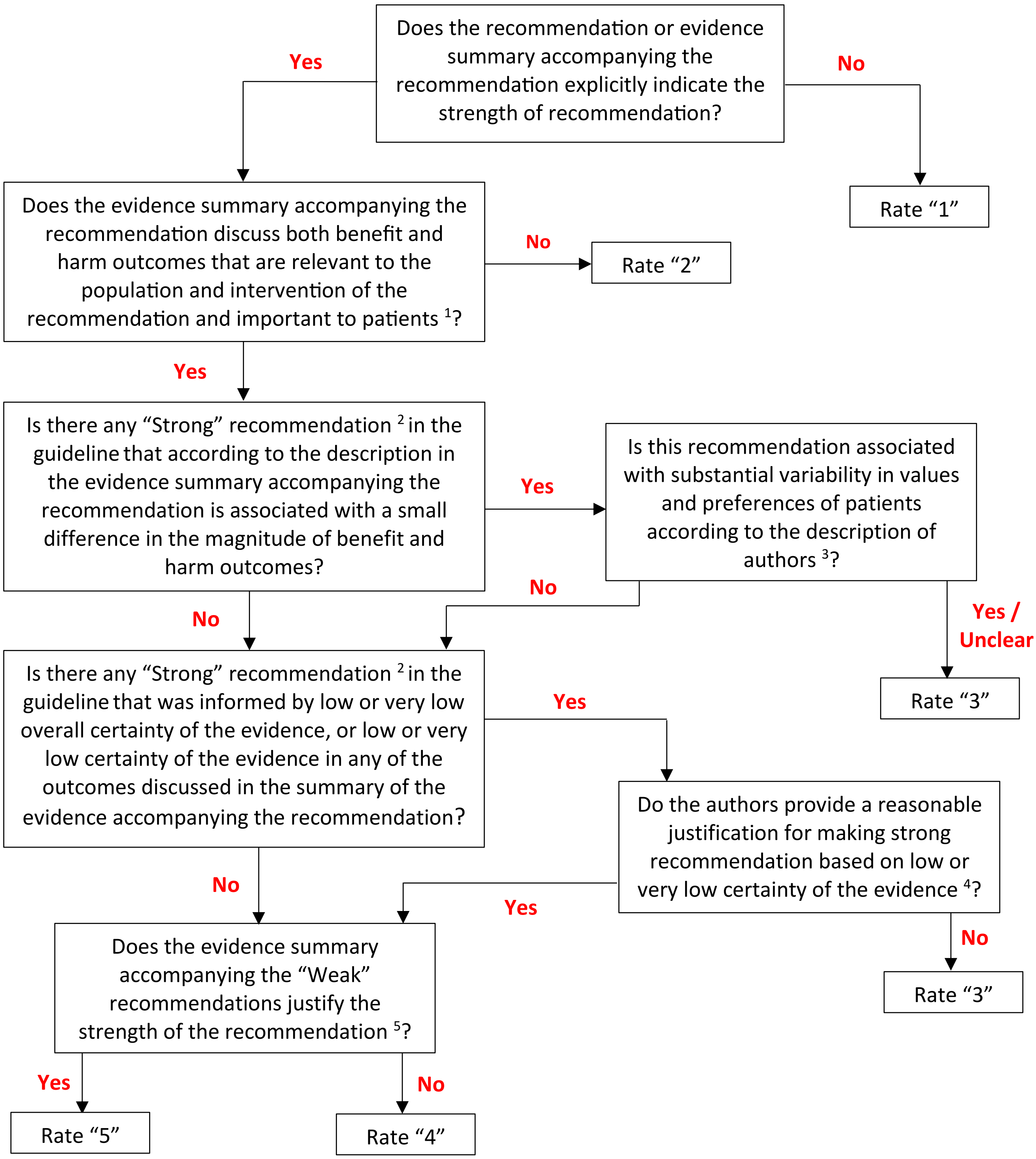

[1] If the guideline acknowledged the lack of evidence on a patient-important outcome and instead used a surrogate outcome to formulate the recommendation, it is acceptable.

[2] Please note that there are different systems for grading the strength of recommendations that label the strength of recommendations differently. For example, a strong recommendation may be labelled as Class A and Class B in the USPSTF system, or Class I (strong) and Class IIa (moderate) in the AHA/ACC system.

[3] Please look for statements on the values and preferences of patients in the evidence summary accompanying the recommendations to answer this question. If there is no description of the values and preferences of patients, please choose "Unclear".

[4] A reasonable justification may include life-threatening situations or catastrophic harms or uncertain benefits and certain risk of harms.

[5] If the guideline does not have any strong recommendations, skip the boxes related to the strong recommendations, i.e., you should answer boxes 1 & 2 & last box. If you reach the last box but the guideline does not have any weak recommendation to assess, rate "5".

6. Specific and Unambiguous Articulation of Recommendations

The recommendations are specific and unambiguous, stating what action should or should not be taken in what situations and for what population groups. Where the recommendations are intentionally vague or underspecified, the guideline clearly describes the rationale behind those recommendations.

Where to Look: Examine recommendations, any infographics or tables that summarized recommendations, and methods for guideline development.

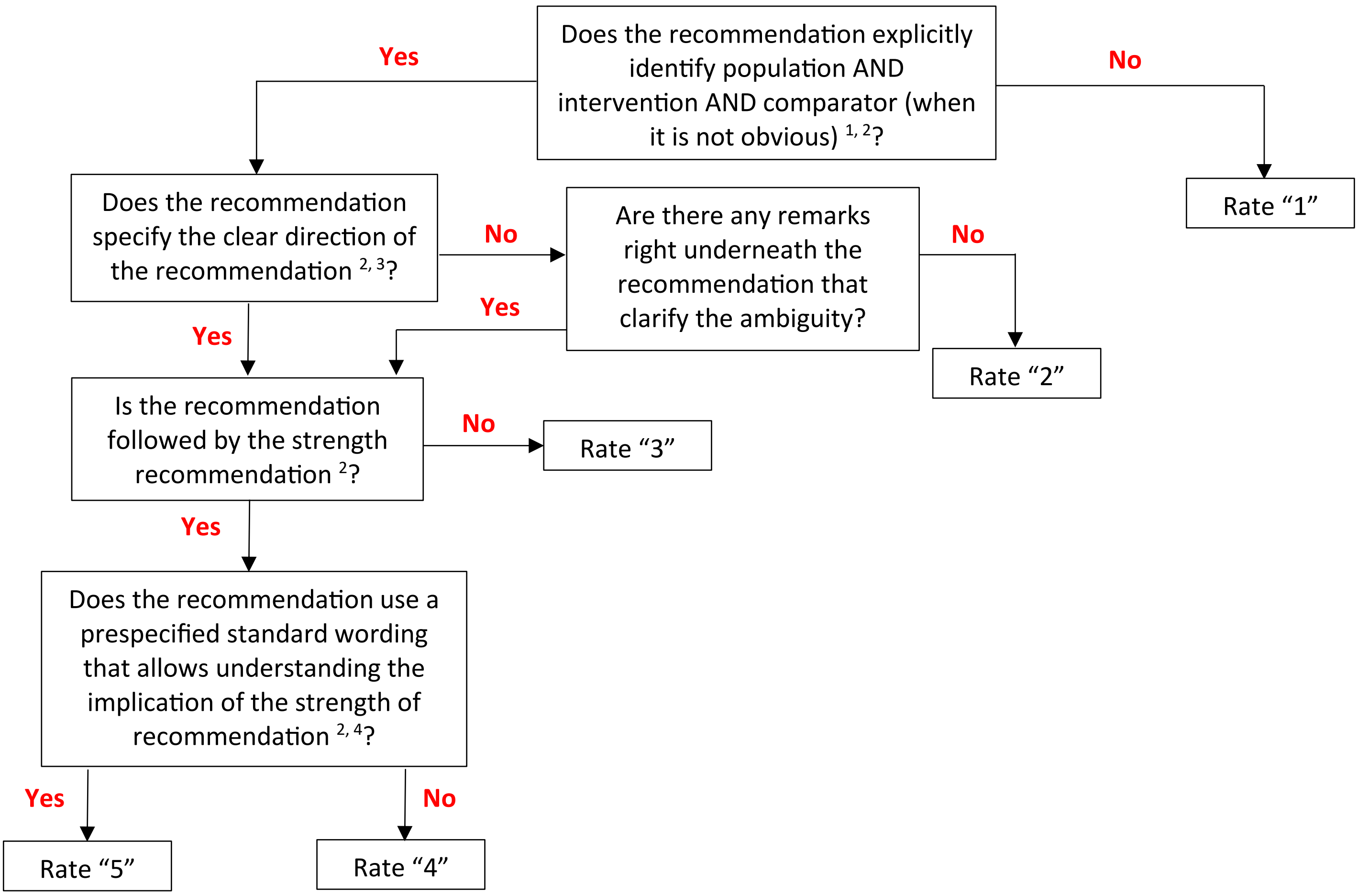

[1] If the comparator is obvious (e.g., we recommend vaccination against COVID-19 in Children 6 months to under 5 years of age who are at high risk for severe illness due to COVID-19; It is obvious that the comparator is no vaccination), it is not obligatory that the recommendation explicitly identifies the comparator.

[2] Please note if recommendations are summarized in any infographics or tables, this criterion needs to be evaluated in these structures as well. To choose "YES" for this question, the criterion must be met in both the recommendation and any associated summarizing structure.

[3] The direction of recommendation clearly presents whether an intervention is recommended (suggested) or not recommended (not suggested) over other intervention(s) like "In patients who have had a stroke, we suggest clopidogrel alone vs. adding aspirin to clopidogrel." Rather than using a vague statement like "In patients who have had a stroke, clinicians should offer clopidogrel alone vs. adding aspirin to clopidogrel, if clinically appropriate."

[4] Standard wording refers to the semantic separation of the strength of recommendations in the wording to convey the implication of the recommendation, for example: for strong recommendation "we recommend…, clinicians should…, clinician should not; and for weak recommendation "we suggest…, we conditionally recommend…, clinicians might…". To choose "YES" for this question: 1) the guideline explicitly specified the standard wording in the methods AND 2) followed the standard wording in the recommendation as specified in the method. So, if any of the 2 criteria are not met, please choose "NO".

7. External review

The guideline has been reviewed by relevant stakeholders, including scientific and clinical experts, organizations, agencies, and patients.

Where to Look: Examine methods for guideline development or guideline development process section, acknowledgement section, contributions section, appendices or any publication platform (like MAGICapp, GRADEpro, or any website, etc.) for information on "Peer review" or "External reviewers".

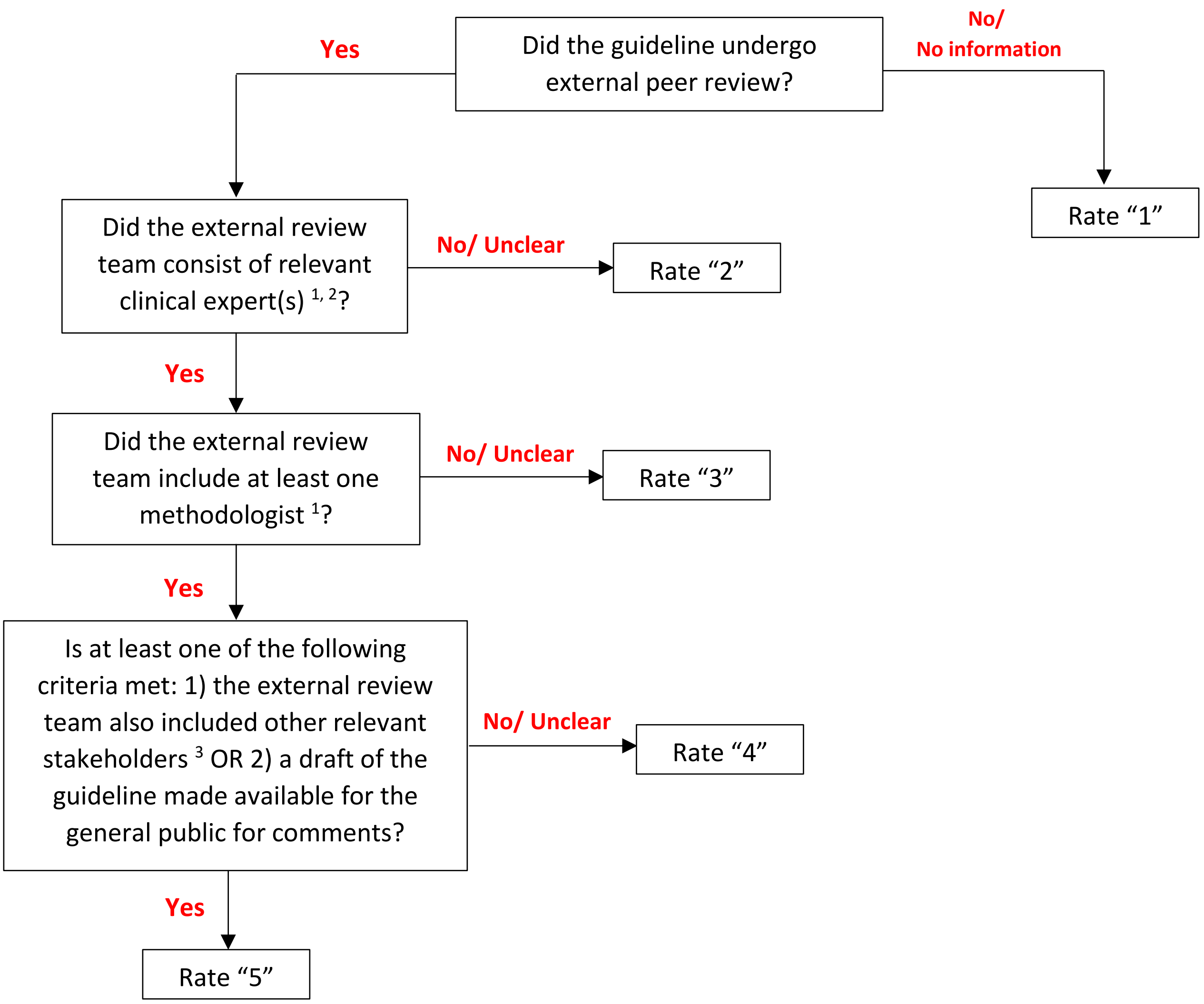

[1] Guidelines may not explicitly list the composition of the external review team. In this case, you must review the affiliations of external reviewers to verify if the discipline was included. If external review team members were not identified or affiliations were not sufficiently specified to confirm the included disciplines, please choose "Unclear".

[2] If a guideline explicitly stated that we included "content experts" in the external review team, you can assume that relevant clinician reviewer(s) were included.

[3] Other relevant stakeholders include patient representatives, representatives from federal agencies, relevant professional organizations or specialty societies, advocacy organizations, public health organizations, health plans, or pharmaceutical or device companies.

8. Updating

The guideline or supporting documents provide an adequate timeframe for the review of evidence for potential updating and describe adequate criteria that warrant updating the recommendation.

Where to Look: Examine methods for guideline development or guideline development process section, appendices, and any publication platform (like MAGICapp, GRADEpro, or any website, etc.) for information on "(future) updates".

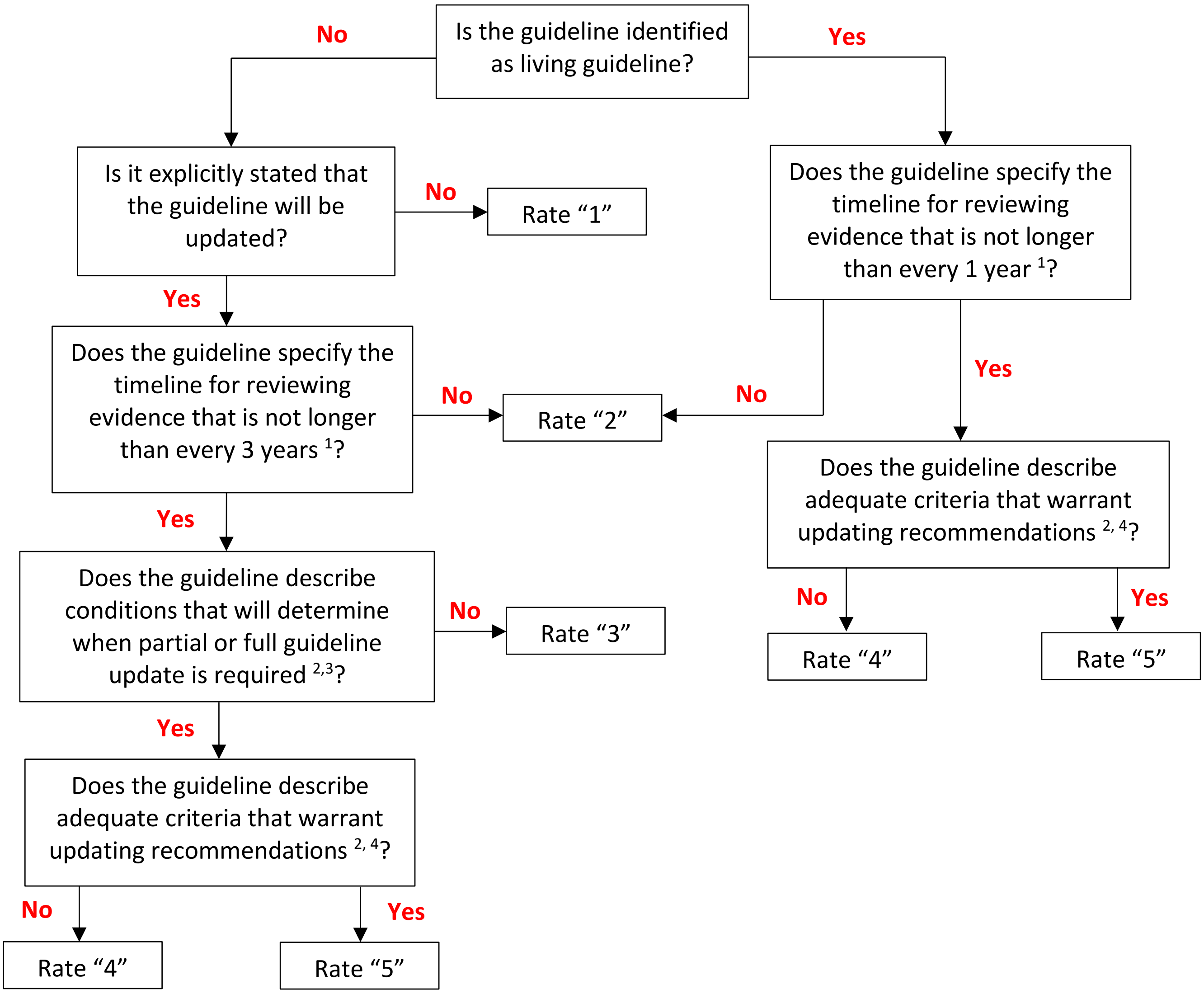

[1] If the guideline states that evidence will be "regularly" monitored without specifying an explicit timeline, please choose "NO" for this question.

[2] Please note that guidelines may not provide detailed information on updating. If the guideline refers to or cites method guidance for the development of guidelines in the guideline development section, please find the full text of the guidance and make sure to review the updating section to see if additional information is provided to help you judge this item. However, the **explicit statement on updating** and the **timeline for updating** must be assessed in the guideline or supplementary documents, not the methods guidance.

[3] For example, a full revision of the guideline is required when at least two previous focused updates and/or new evidence suggests that a significant number of recommendations require revision.

[4] For example, a guideline should be updated if new evidence shows that a recommended intervention causes previously unknown substantial harm; that a new intervention is significantly superior to a previously recommended intervention from an efficacy or harms perspective; or that a recommendation can be applied to new populations.
